# Associating conditional cash transfer to universal access to treatment could be the solution to the HCV epidemic among drug users (DUs)

**DOI:** 10.1186/s12954-018-0264-4

**Published:** 2018-12-12

**Authors:** Philippe Chossegros, Fiorant Di Nino

**Affiliations:** 1 UHSI de Lyon, Centre Hospitalier Lyon SUD, Hospices Civils de LYON, Chemin du Grand Revoyet, 69495 Pierre-Bénite, France; 2 Association ITHAQUE, 12 rue Kuhn, 67000 Strasbourg, France

**Keywords:** Drug use, Respondent-driven sampling, HCV, Epidemic, Social networks

## Abstract

**Background:**

To understand the limits of HCV screening programs to reach all drug users (DUs).

**Method:**

The association of the recruitment of a representative sample of a population of DUs in a specific area with the use of a questionnaire that included 250 items allowed the use of uni- and multifactorial analysis to explore the relationship between HCV screening and dimensions until now restricted to qualitative studies.

**Results:**

We recruited, in less than 2 months, 327 DUs representing about 6% of the total population of DUs. They belonged to a single community whose drug use was the only common characteristic. While almost all DUs (92.6%) who had access to care providers had been screened, this proportion was much lower in out-of-care settings (64%). HCV prevalence among those who had performed a test was low (22.8%). For DUs, the life experience of hepatitis C has not changed in the last 10 years. Screening, studied for the first time according to this life experience, was not influenced by a rational knowledge of the risk taken or the knowledge of treatment efficacy, showing a gap between DUs’ representations and medical recommendations which explains the low level of active screening. Police crackdown on injections, disrupting the previous illusion of safe practices, was the only prior history leading to active screenings. Screenings were related to an access to care providers. GPs held a preponderant position as a source of information and care by being able to give appropriate answers regarding hepatitis C and prescribing opioid substitution treatments (OST). If 48 % of DUs screened positive for HCV had been treated, half of them had been prescribed before 2006.

**Conclusion:**

While hepatitis has become a major issue for society and, consequently, for services for DUs (SDUs) and GPs, it is not the case for DUs. A widespread screening, even in a city where the offer of care is diversified and free, seems unlikely to reach a universal HCV screening over a short time. The model of respondent-driven sampling recruitment could be a new approach to conditional cash transfer, recruiting and treating DUs who remain outside the reach of care providers, a prerequisite for the universal access to HCV treatments to impact the HCV epidemic.

## Background

Viral infections of DUs and more particularly hepatitis C [1] are recognized as a public health problem. Hepatitis B vaccination prevents new HBV infections [2], antiviral treatments of hepatitis B [3, 4] and AIDS [5] control the evolution of these diseases, and direct-acting antivirals (DAA) make it possible to cure almost all hepatitis C [6]. Despite their effectiveness, their impact on the course of these epidemics will not be fully realized as long as patients are left untreated [7, 8]. A universal screening is a prerequisite to succeed [9].

Due to the illegal nature of drug use, part of the population of DUs remains hidden. Understanding the course of this epidemic suffers from variation in the coverage and quality of existing research and, most of all, of convenience sampling which may be responsible for representative bias [10, 11]. This lack of reliable data has been emphasized by most international reviews [12–14] as well as French recommendations [15]. More precise data on coherent territories are needed. Different approaches have been proposed to recruit a representative sample of DUs active in a given territory.

Apart from the use of drugs, DUs differ from the general population by their socio-economic situation and by their particularly high risks of contamination by infectious agents. Although HCV prevalence has been frequently studied, other elements such as the influence of their use of drugs, their social networks, their fears, their projects, their sources of information, and their opioid substitution treatment on their access to screening have been little studied. In 2008, Treloar and Rhodes, reviewing 31 former papers in a qualitative meta-ethnographic analysis of the social production of hepatitis risk, concluded that, for injecting DUs, HCV hepatitis is a liminal illness, a risk accepted rather than avoided and that symbolic knowledge systems, rather than biomedical risk calculus, and especially narratives of hygiene and trust, played a primary role in shaping interpretations of HCV risk [16]. However, non-injectors were excluded, and they did not analyze the relationship of this life experience with HCV screening. The most recent studies were only conducted among intravenous DUs (IVDUs) attending primary and secondary care services. Strauss et al. found a low knowledge of hepatitis C among injectors and non-injectors who were unaware of the existence of HCV education opportunities [17]. Barocas et al., in Southern Wisconsin, found that access to healthcare was an important determinant of regular HCV screening, indicating that self-initiated screening was less successful than provider-initiated HCV screening, consistent with a previous study conducted in San Francisco and New York [18, 19]. For Swan et al., HCV was considered as a relatively benign infection, competing with other priorities. Difficulties in accessing specialized care and treatment were improved by trust in providers and education on HCV infection [20]. Walley et al. “interested” attendants of a methadone clinic in HCV treatment after an education program [21].

In 2011, we decided to improve the quality of our care by a better knowledge of DUs. First, in a partnership between professionals and DUs attending services for DUs (SDUs), we wrote a questionnaire including all relevant information that would help to understand DU’s life (Table 1). Then, the same year, we used it to study DUs recruited by a snowball sampling initiated in all the SDUs of the greater Strasbourg. Nobody was paid. If the content of the questionnaire has been validated by its analysis, the snowball recruitment was a failure, since it was only able to recruit 145 DUs for a period of 1 year with 2 DUs outside of the health care system. This failure led us to consider another method to recruit a representative sample of Strasbourg’s population of DUs.

**Table 1:** Items covered in the questionnaire

Sex Year of birth (S) Country of origin (N) Education (N): primary school, college, bachelor’s degree, university, master Housing (N): owner, tenant, parents, friends, shelter, homeless Work (N): permanent and fixed term contract, studies, unemployment, pension, none Source of income: employment, unemployment, income support, disability, illegal, none (N) Health insurance: mutual health insurance, general, universal health-care coverage, adult disablement allowance, none, State Medical Aid (N) Drugs (start date (S), life and current use (N)): heroin, cocaine, alcohol, misused opiate substitution therapy (MOST), amphetamine and other stimulants, ecstasy, ketamine, LSD and hallucinogen, benzodiazepine, codeine, cannabis Drug use (life long, current) (N): alone, with a partner, with a friend, in a group Conviviality of the use of drugs (N): alone, with one’s partner, with friends or in a group Type of use (N): sharing of needles and syringes (NSS) and its level, sharing of drug injection paraphernalia, sharing of straws, sharing of pipes (crack) Sharing at a time of craving (N): NSS, drug paraphernalia, straw, pipe Access to sterile (N): needle and syringes, drug paraphernalia, straw and pipe Source of syringes and drug paraphernalia: (N) pharmacy, UDs’ service, bus, entourage Opiate substitution therapy (OST): methadone, buprenorphine, morphine sulfate (current and past prescription, start date, injection) Police crackdown on injections and opiate substitution (N) HCV (N): screening (presence and date), serology (result and date), reasons invoked so as not to be screened and treated, knowledge of treatment efficacy, treatment, HCV immediate screening acceptance HBV (N): screening (presence and date), serology (result and date), knowledge of vaccination and treatment efficacy and side effects, vaccination, treatment HIV (N): screening (presence and date), serology (result and date), treatment Sources of information on health (NB): friends, television, press (free and paid-for), Internet (computer and smartphone) and social networks, GP, DUs’ service, pharmacy, festive stand of prevention, leaflet Life plans (NB): Social: find housing, change housing, pay one’s debts, find a job, change job, be entitled to social security Health: taking care of one’s teeth, making a health check, screening for hepatitis, treating one’s hepatitis, vaccination, treating skin ulcers Drug: decrease drug use, control drug use, stop drug use, decrease treatment Circle: found a family, come out of isolation, do an activity, see one’s family, find a companion Fears (NB): of stopping drug use, of not being able to stop drug use, of overdoses, of going to jail, of not being able to pay one’s debts, of contamination, of losing one’s job, of losing one’s friends, of losing the custody of one’s children, of losing contact with one’s group, of staying alone Social networks (number met during the last 2 weeks (S)): parents, friends, partner, co-workers, healthcare professionals, drug users, dealers Sexual preference (N) Number of sexual partners (last 6 months) (S) Use of condoms (last 6 months) (N) Care (start date, current and past)(NB): low threshold services, drug treatment center, GPs, bus	

*S* scale, *N* nominal, *NB* nominal binary

Respondent-driven sampling (RDS) is currently one of the most popular. It has been applied in more than 460 peer-reviewed surveys conducted in 69 countries throughout the world [22]. It is used by the Centers for Disease Control and Prevention to track the HIV epidemic [23, 24]. Yet, studies that have applied this method to DUs have been almost constantly limited to risk behaviors, search for viral prevalence, and access to care.

This RDS study was funded in 2015 through a grant of the “Fonds de dotation Gilead Sciences.”

We present the analysis of this database from the perspective of HCV screening, contamination, and treatment. To our knowledge, it is the first study to focus, in a representative sample of DUs in a well-defined area, on the relation between HCV screening history and the elements that may have influenced this past decision or that could lead to further screening and treatment, information that could help build a more effective strategy to eradicate hepatitis C among DUs.

## Methods

### Study design

Our study was conducted in the greater Strasbourg area (15–64 years, 473,375 people). RDS was chosen to recruit a representative sample of DUs. The condition of their eligibility followed the European Monitoring Centre for Drugs and Drug Addiction definition of problematic drug use: injecting drug use or long duration/regular use of opioids, cocaine, and/or amphetamines [25]. Non-injectors were included because most injectors begin as non-injectors, because HCV prevalence in this population has been shown to be much higher than in a non-drug using population [26], and because we wanted to compare non-injectors to injectors in their relation to screening. Strauss et al. considered critical for prevention that non-injectors understand the HCV related risks involved in sharing both injection and non-injection drug use equipment [17].

We started with 3 “seeds” randomly recruited among DUs attending the same SDU. They were asked the number of DUs they had met in the previous 14 days (network size) and were given 3 coupons to recruit 3 respondents among their own social network who were due to show up less than 14 days later. Each wave of respondents, following the same rules, recruited the next wave until the final sample size was achieved. Our budget allowed financing the recruitment of 300 DUs representing a sample of 6% of the total population of UDs of the greater Strasbourg area [27].

The completion of a 250-item auto-questionnaire by each respondent was followed by a cross-examination of their answers in a private space by the same professional interviewer to assessed their quality and completeness. HCV serostatus was categorized according to participants’ self-report. No one had to perform a new HCV screening. The monetary recruitment incentive was 30 € for the completion of the interviewer-administered questionnaire and 20 € for each recruited UD. This amount was considered to be a fair salary, recognizing an actual work, by the DUs who had validated the questionnaire. Since giving money unconditionally regarding its use to homeless and other low-income individuals, among which DUs, has constantly been shown to improve their empowerment, this approach was considered ethical [28–30]. The conduct of the study was in line with the ethical checklist (respect, beneficence, justice, and safeguards) provided by Semaan and al. [31].

Patients provided informed consent to participate in the study and to publish its results. This study was approved by the French Commission Nationale de l’Informatique et des Libertés (CNIL).

### Statistical analysis

Data were anonymized before analysis. RDS allows statistical adjustments for differential network sizes to produce estimates representative of the sampled population’s network. RDS data weighting was used to compare the observed distribution of HCV screening between the observed and the weighted population as well as their homophily for sex and age using RDSAT version 7.1.46: (http://www.respondentdrivensampling.org/reports/RDSAT_7.1-Manual_2012-11-25.pdf; http://www.respondentdrivensampling.org/download/RDSATv7.1.46/RDSAT_windows-x64_7_1_46.exe). Equilibrium was calculated for participant’s age, sex, injection, HCV screening, and HCV prevalence.

The items considered pertinent to understand HCV screening (HCV screening, HCV contamination, knowledge of the efficacy of HCV treatment, plans for the future, fear of contamination) were compared to all the other variables. For ordinal or categorical variables, chi-square and odds ratio or Kendall tau-b were calculated. *t* test or one-way ANOVA was performed for scale variables. Since multiple comparisons were performed, statistical significance was considered at an alpha of 0.01 to prevent the occurrence of too many statistically but not clinically relevant associations. To measure the association between dependent and independent variables, the significant variables were entered into binary logistic regression multivariate models and automatically chosen by forward stepwise selection procedure (two sides, *p* < 0.05). Odds ratios (OR) and 95% confidence intervals (CI) were calculated from *β* coefficients and their standard errors. The Hosmer-Lemshow test was used to determine the goodness of fit of the models. The data was analyzed using IBM SPSS version 19.

## Results

### Respondent-driven sampling and characteristics of drug users

Recruitment started with 3 seeds: 2 men (1 low threshold service, 1 drug treatment center) and 1 woman (needle and syringe exchange program). The first chain recruited 117 UDs and was interrupted at the sixth wave. The second and third chains included 96 and 114 DUs, respectively, and were interrupted at the fourth wave. A total of 327 UDs were recruited in less than 2 months (February to March 2015). All the participants enrolled met the eligibility criteria. None of the 3 chains was interrupted for lack of new respondents. The mean number of DUs encountered by each respondent during the 15 days preceding the interview was 13.6 (95% CI 11.6–15.5). Whatever item is chosen, a tendency toward in-group recruitment coexisted with considerable cross-group recruitment, but no barrier was observed between the groups as shown in Table 2. Sex was the only characteristic demonstrating a negative homophily.

**Table 2:** Homophily for sex, age, intravenous drug use, and HCV screening and serology

	Homophily
Male	0.093
Female	− 0.137
Age	
< 20	0.198
20–29	0.08
30–39	0.114
40–49	0.073
> 50	0.054
Non-IVDU	0.135
IVDU	0.118
HCV screening+ HCV+	0.159
HCV screening+ HCV−	0.125
HCV screening−	0.154

Homophily refers to the tendency to recruit in the same category with a metric between 1 (recruited and recruits in the same category) and − 1 (recruited and recruits in a different category). Heckathorn suggests any value of homophily ≥ 0.3 as intermediate homophily and any value ≤ − 0.3 as strong heterophily

There was no statistical difference between the observed and weighted distribution of screening compared to injection (Table 3).

**Table 3:** Observed and weighted distribution of the cross-tabulation of screening by injection

Screening\Injection	Yes, 95% CI	No, 95% CI
Observed		
Yes	54.1, 48.6–59.6	7.6, 4.8–105
No	27.8, 23–33	10.4, 7.3–14.2
Weighted		
Yes	50.8, 42.5–58.8	9.8, 5.3–16.6
No	27.8, 20.8–33.1	11.6, 7–17.6

The mean age of our sample was 33.4 years (95% CI 32.3–34.4). Mean age of the first drug experimentation was 15.02 years. Age of the first injection was 21 years (95% CI 20.25–21.67). The mean number of drugs consumed during life was 8.66 (95% CI 8.39–8.94). Socio-economic and drug-related characteristics of the participants are presented in Tables 4 and 5.

**Table 4:** Socio-professionnal characteristics of DUs (semiquantitative)

	Observed nb	Percent	95% CI
Sex			
Men	243	74.3	69.6–79.0
Women	84	25.7	21.0–30.4
Nationality			
French	275	84.1	80.1–88.1
Foreigner	52	15.9	11.9–19.9
Housing			
Stable (owner or rent)	141	43.1	37.9–48.5
Parents	38	11.6	8.6–15.6
Friends	22	6.7	4.5–10.0
Shelter	37	11.3	8.3–15.2
Homeless, squat	89	27.2	22.7–32.3
Education			
Primary	10	3.1	1.7–5.5
Secondary	225	68.9	63.6–73.6
Bachelor	52	15.9	12.3–20.3
Higher education	40	12·2	8.7–15.8
Home			
Parents	49	15	11.1–18.9
Couple	103	31.5	26.5–36.5
Alone	145	44.3	38.9–49.7
Other	30	9.1	6.0–12.2
Profession			
Studying	10	3.1	1.0–5.0
Permanent contract	32	9.8	6.6–13.0
Fixed term contract	38	11.6	8.1–15.1
Unemployed	58	17.7	13.6–21.8
Disability	9	2.8	1.0–4.6
Pension	1	0.3	−0.3-0.9
None	179	54.7	49.3–60.1
Active solidarity income	150	45.9	40.6–51.3
Prison	162	49.5	44.08–54.92
Sexual preference			
Heterosexual	295	90.2	85.5–93
Bisexual	26	8	5.5–11.4
Homosexual	6	1.8	0.84–3.9

**Table 5:** Characteristics related to drug use

	Observed number	Percent	95% CI
History of injection			
NSS+	114	34.9	29.9–40.2
NSS−	89	27.2	22.7–32.3
None	125	38.2	32.9–43.5
Treatment for drug use			
Never	80	24.5	19.5–28.7
Current	202	61.8	56.5–67.1
Given up	45	13.9	10.1–17.5
Drug treatment center	59	28.7	23.32–33.08
Low threshold services	26	12.9	9.27–16.53
Bus	23	11.4	7.96–14.84
GPs	141	69.8	64.82–74.78
Current opiate substitution therapy			
Methadone	85	26	21.3–31.1
Buprenorphine	65	19.9	15.7–24.6
Morphine sulfate	15	8.6	2.6–7.5
Fears			
Prison	161	49.2	43.9–54.6
Not stopping drug use	146	44.6	39.4–50.0
Contamination	143	43.7	38.5–49.2
Staying alone	138	42.2	37.0–47.6
Losing one’s friends	117	35.8	30.8–41.1
Overdose	91	27.8	23.3–32.9
Not paying one’s debts	74	22.6	18.4–27.5
Stopping drug use	69	21.1	17.0–25.9
Losing custody of children	44	13.5	15.6–24.2
Losing contact with friends	34	10.4	7.54–14.2
Losing one’s job	32	9.8	7.0–13.5
Sources of health information			
GP	180	55	49.6–60.4
Friends	121	37	32.0–42.4
SDU	90	27.5	23.0–32.6
Internet (computer)	89	27.2	22.7–32.3
Television	86	26.3	21.8–31.3
Booklet	85	26	21.5–31.0
Stand	50	15.3	11.8–19.6
Family	46	14.1	10.7–18.3
Press (free)	42	12.8	9.6–16.9
Internet (smartphone)	26	8	5.5–11.4
Social networks	25	7.6	5.2–11.0
Press (paid for)	23	7	1.7–10.3
HCV screening			
Adequate	241	73.7	68.7–78.2
Inadequate	26	7.95	5.5–11.4
Absent	60	18.3	15.5–22.9
Positive	61	22.8^a^	18.2–28.3
Negative	206	77.2^a^	71.8–81.8
Unknown	60	18.4^b^	14.5–22.9

*NSS* needles and syringes sharing, *GP source* GP as source of health information, *none* no injection, *SDU* service for drug users, *SDU source* service for drug users as source of health information, *Source of information* source of health information, *HCV screening* adequate less than 1 year or absence of injection since the last test, *Inadequate* current injector with more than 1 year since the lest test, *absent* no test

^a^Prevalence among screened DUs

^b^Prevalence among the 327 DUs

### HCV screening

Testing was not related to any socio-demographic characteristic such as sex, nationality, education, profession, social coverage, or housing. If friends were the second most frequent source (37%) of health information after GPs (55%) and before SDUs (27.5%), their opinion did not influence HCV screening. No specific plan for the future was associated with a previous test. There was no difference between DUs whose screening result was positive or negative.

HCV screening was associated with age (screening + 35.01 (95% CI 33.95–36.08)/screening − 25.90 (95% CI 23.33–28.46), *p* < 0.0001) as well as duration of drug use (screening + 19.46 (95% CI 18.43–20.49)/screening − 11.37 (95% CI 8.76–13.99), *p* < 0.0001) and duration of injection (screening + 14.79 (95% CI 13.14–16.44)/screening − 2.87 (95% CI 1.60–4.14), *p* < 0.0001). The results of the other significant univariate tests and multivariate logistic regression models measuring the association of past year HCV testing and selected participant characteristics are presented in Table 6.

**Table 6:** Factors significantly associated with an HCV test in univariate tests and binary logistic regression (forward stepwise selection)

HCV screening	% screening	% total	Sig	Odds ratio	95% CI odds ratio	
					Lower	Upper
Injection			0.002	0.378	0.213	0.672
Yes	87.2	62.1				
No	73.4	37.9				
NSS			< 0.001	5.363	2.476	14.358
Yes	94.7	34.9				
No	75.1	65.1				
Craving			< 0.0001	4.392	1.819	10.606
Yes	93.7	29.1				
No	77.2	70.9				
Police			< 0.0001	9.344	3.257	26.807
Yes	96.3	38.3				
No	73.6	61.7				
GP source			< 0.0001	5.231	2.735	10.005
Yes	92.2	55.0				
No	69.4	45.0				
SDU source			0.008	3.810	1.276	6.188
Yes	91.1	27.5				
No	78.5	72.5				
SDU or GP			< 0.0001	6.772	3.566	12.861
Yes	92.6	61.8				
No	65.7	38.2				
Prison			< 0.0001	7.833	3.555	17.261
Yes	94.7	49.5				
No	69.7	50.5				
Logistic regression	*B*	Wald	Sig	Odds ratio	95% CI odds ratio	
					Lower	Upper
All: 327 cases						
Age	0.099	12.764	< 0.0001	1.100	1.044	1.159
Police	− 1.338	7.060	0.008	0.262	0.098	0.704
OST	− 1.750	9.311	0.002	0.174	0.057	0.535
GPsource	− 1.014	6.347	0.012	0.362	0.165	0.798
Prison	− 1.073	4.748	0.029	0.342	0.130	0.898
Constant	1.593	2.051	0.152	4.921		

Hosmer and Lemeshow test, 0.101; CC—screening+, 94.8%; screening−, 44.8%; all, 85.8%

Logistic regression odds ratios—police: absent/present, injection: NSS− and no injection/NSS+, OST: absent/present, GP source: absent/present

*Injection* history of injection, *NSS* needle and syringe sharing, *Craving* NSS at a time of craving, *Police* police crackdown on injections, *GP source* GP as source of health information, *SDU* service for drug users, *GP* medical practice of a general practitioner, *OST* opiate substitution therapy, *prison* having served a prison sentence, *CC* correctly classified

Multiples reasons (54/60) were given by respondents to explain the absence of screening. An absence of concern (85.2%, 95% CI 73.4–92.3) came first, followed by problems with blood access (22.2%, 95% CI 13.20–34.94). A fear of the result was mentioned by seven, and only three reported being unaware of voluntary testing sites. However, two thirds (61.3%) accepted the theoretical proposal for immediate screening, significantly lower (86%) than those who had previously been screened (odds ratio 0.26, 95% CI 0.11–0.61, *p* < 0.001).

HBV (67.8%, 95% CI 67.79–72.63), HIV (86.8%, 95% CI 82.70–90.06), and HCV screening were very closely linked (HBV/HCV odds ratio 36.52, 95% CI 14.90–89.55; HIV/HCV odds ratio 23.53, 95% CI 10.87–50.92) and were also linked to the same items (duration of drug use, access to treatment of drug use and, for HBV, police crackdown on injections).

### HCV contamination and treatment

HCV prevalence (observed 22.8%, weighted 21%) was low. In a univariate analysis, HCV contamination was related to the risks related to drug use such as its duration (HCV − 17.9 years, 95% CI 16.8–22.9/HCV+ 24.9, 95% CI 22.9–26.9), needle and syringe sharing (NSS), episodes of craving or police crackdown on injections, and history of care; once the level of risks and the duration of drug use were taken into account, care disappeared because they were strongly related to age and duration of drug use (Table 7). Sharing of works was not an additional risk of contamination. Access to sterile works was considered easy by 88.7% of current injectors who used syringe-dispensing machine, needle exchange program, bus, and pharmacy at the same time. It was not related to HCV screening or contamination. The high odds ratio associated with OCT is explained by this correlation.

**Table 7:** Factors significantly associated with an HCV positive (HCV+) test in univariate tests and binary logistic regression (forward stepwise selection)

HCV+	% HCV+	% total	Sig	Odds ratio	95% CI odds ratio	
					Lower	Upper
Injection			< 0.0001	22.250	5.294	93.518
Yes	33.3	66.3				
No	3.3	33.7				
NSS			< 0.0001	15.579	7.205	33.688
Yes	48.1	40.3				
No	5.6	59.7				
Craving			< 0.0001	9.26	4.83	4.83–17.74
Yes	49.4	33.2				
No	9.5	66.6				
Police			< 0.0001	3.519	1.858	6.663
Yes	36.5	44.6				
No	14.1	55.2				
SDU or GP			< 0.0001	0.191	0.100	0.366
Yes	29.9	69.8				
No	6.2	30.2				
OST			< 0.0001	4.959	2.444	10.060
Yes	29.8	67.8				
No	8.1	32.2				
SDU			< 0.0001	3.420	1.814	6.450
Yes	42.1	21.3				
No	17.5	78.7				
Prison			0.009	2.279	1.212	4.274
Yes	29.9	57.4				
No	15.7	42.6				
Logistic regression	*B*	Wald	Sig	Odds ratio	95% CI odds ratio	
					Lower	Upper
NSS	0.108	17.440	< 0.0001	1.115	1.080	1.174
Drug use	− 2.623	33.403	< 0.0001	0.73	0.30	0.177
Police	− 1.190	8.310	0.004	0.323	0.150	0.697
Constant	− 1.939	8.310	0.002			

Hosmer and Lemeshow test, 0.991; CC—HCV+, 51.8%; HCV−,90.3%; all, 81%

*Injection* history of injection, *NSS* needle and syringe sharing, *Craving* NSS at a time of craving, *Police* police crackdown on injections, *SDU* service for drug users, *GP* medical practice of a general practitioner, *OST* opiate substitution therapy, *Drug treatment center* attending SDU or GP, *Drug use* duration of drug use, *CC* correctly classified

Logistic regression odds ratios: NSS absent/present, police: absent/present, injection: NSS− and no injection/NSS+, craving: injection at a time of craving−/injection at a time of craving+ (267 cases: exclusion of HCV+ DUs)

A spontaneous clearance of HCV was reported in 11 cases (18%, 95% CI 10.4–29.5). Among those who had not spontaneously cleared HCV, 23 had been treated (46%, 95% CI 33–59.6, mean year 2005, range 1990–2015). Treatment had been completed in 21 cases, and 13 believed they had been cured. The decision to be treated was favored in 14 cases (60.9%) by trust in the specialist or in the medical team. Among the 27 who had not been treated, the main reason was an absence of severity of hepatitis (59.2%). The main cause of patients’ refusal was side effects (33.3%), followed by not feeling ill (25.9%) and by problems with venous access (22.2%). Alcohol was presented as preventing treatment for doctors (2 cases) as well as for patients (6 cases).

HBV (5.3%, 95% CI 2.99–9.26) and HIV (1.7%, 95% CI 0.75–4.03) were too low for a statistical analysis. Two of the 11 HBV-positive and 2 of the 5 HIV-positive DUs had never injected.

### Fear of contamination

Fear of contamination was neither related to any previous risk of contamination (drug or condom use) nor to the duration of drug use or a previous HCV test. It was associated with planning a future HCV test that did not increase the rate of acceptance of an immediate screening proposal. Among the factors associated with fears of contamination, other fears (total number and fear of overdoses) came first (Table 8). For once, socio-demographic characteristics were concerned with a protection for DUs who worked with social security coverage and a stable housing and an increased prevalence among DUs with illegal incomes.

**Table 8:** Factors significantly associated with fear of contamination in univariate tests and binary logistic regression

Fear of contamination	% fear	% total	Sig	Odds ratio	95% CI odds ratio	
					Lower	Upper
Univariate						
Health			0.009	1.937	1.179	3.182
Yes	52.7	41.4				
No	36.5	56.6				
Hepatitis			< 0.0001	5.419	1.947	15.080
Yes	78.3	8.6				
No	39.9	91.4				
Overdose			< 0.0001	5.292	2.936	9.537
Yes	71.9	27.8				
No	32.2	72.7				
Illicit*			0.004			
Legal	35.4	55.3				
Moonlighting	43.9	18.9	NS	1.42	0.73	2.76
Illegal	59.2	26.7	< 0.001	2.65	1.18	4.74
Social inclusion			< 0.0001	0.11	0.03	0.375
Yes	9.1	12.4				
No	48.1	87.6				
Logistic regression	*B*	Wald	Sig	Odds ratio	95% CI odds ratio	
					Lower	Upper
Fears Nb	0.580	27.333	< 0.0001	1.751	1.419	2.160
Social inclusion	− 1.851	6.347	0.012	0.157	0.037	0.863
Hepatitis	− 1.766	7.881	0.005	0.171	0.050	0.587
overdose	− 0.939	6.047	0.014	0.314	0.185	0.827
Illicit*						
Legal	− 0.973	6.673	0.010	0.378	0.181	0.791
Moonlighting	− 1.120	4.745	0.029	0.391	0.185	0.827
Constant	2.933	6.552	0.010	18.790		

Hosmer and Lemeshow test, 0.777; CC—Fear+, 71.9%; Fear−, 85.1%; all, 79.9%

Logistic regression odds ratios: social inclusion (forward stepwise selection) (266 cases: exclusion of HCV+ DUs)

*Fears Nb* total number of fears, *Health* plan for a health check, *Hepatitis* plan for HCV screening, *Overdose* fear of overdoses, *Illegal* drug trafficking or theft, *Social inclusion* having a job, housing, social security

### Knowledge of the efficacy of HCV treatments

An accurate knowledge of the efficacy of HCV treatments (37.9%; OR CI 32.6-43.4) was higher among seropositive DUs (+ 72.1/others 30.2%; odds ratio 95% CI 3.23–11.14; *p* < 0.0001). The knowledge of the efficacy of HBV and HCV treatments and of HBV vaccination were higher among HCV-positive respondents than HCV-negative ones. Among seronegative cases, GPs were the only source of accurate knowledge (Table 9).

**Table 9:** Factors significantly associated with the knowledge of the efficacy of HCV treatment in univariate tests and binary logistic regression (forward stepwise selection) among HCV negative respondents

HCV treatment	% treatment	% total	Sig	Odds ratio	95% CI odds ratio	
					Lower	Upper
Univariate						
GP source			0.001	2.545	1.473	4.395
Yes	39.6	50.4				
No	20.5	49.6				
GP			< 0.0001	2.919	1.700	5.009
Yes	43.5	40.6				
No	20.9	59.4				
HBV treatment			< 0.0001	3.770	2.176	6.531
Yes	48.0	36.8				
No	19.6	63.2				
HBV vaccine	49.6		< 0.0001	3.377	1.928	5.915
Yes	42.4	49.6				
No	17.8	50.4				
Logistic regression	B	Wald	sig	Odds ratio	95% CI odds ratio	
					Lower	Upper
GP	− 0.853	8.499	0.004	0.426	0.240	0.758
GP source	− 0.648	4.720	0.030	0.523	0.291	0.939
Constant	− 0.088	0.176	0.675	0.916		

Hosmer and Lemeshow test, 0.104; CC—Knowledge+, 0%; Knowledge−, 100%; all, 69.9%

Logistic regression odds ratios: GP absent/present, GP source: absent/present (266 cases: exclusion of HCV+ DUs)

*HCV treatment* accurate knowledge of the efficacy of HCV treatment, *GP source* general practitioner as source of health information, *GP* medical practice of a general practitioner, *HBV treatment* knowledge of the efficacy of HBV treatment, *HBV vaccine* knowledge of the efficacy of HBV vaccine

### Plans for the future

Looking for a job (62.4%; 95% CI 56.9–67.7) was the main concern among the 28 possible projects. It was followed by taking care of one’s teeth (58.7%; 95% CI 53.2–64.1), returning to activity (42.8%; 95% CI 37.4–48.4), reducing drug use (40.7%; 95% CI 35.3–46.2), and making a health check (40.4%; 95% CI 35–45.9). Making a health check was unrelated to age, NSS, HCV screening, and serology results as well as seeking health information from GPs or DUs’ services. A more specific project of HCV screening (11.9%; 95% CI 8.6–15.9) and engaging in one’s hepatitis treatment (5.8%; 95% CI 3.5–8.9) ranked 25th and 28th, respectively. The only item significantly associated with plans of a future screening for hepatitis was the fear of contamination.

## Discussion

This study, the first to use the RDS method to recruit DUs in France, recruited a population younger and with lower HCV prevalence than those of other French studies that included IV- and non-IVDUs [32–34] because of the inclusion of users left outside treatments for drug use (38.2%) who were previously not studied. Compared to the previous snowball sampling, RDS was significantly superior to recruit this hidden population (OR 115.4, 95% CI 28.12–474.81) The interviews confirmed Heckathorn’s initial hypothesis that money was not the only reason for participating in the investigation [35]. Respondents were eager to have the opportunity, for once, to tell the story of their lives. All these DUs belonged to a diverse community whose only common feature was drug use with no barriers between injectors and non-injectors. HCV prevalence was low (overall 22.8%, IVDUs 33.3%), and HIV and HBV prevalence (5.3 and 1.7%) were too low to allow any statistical analysis.

Our results have points of divergence and concordance with respect to previous studies on the same subject.

1. The main difference concerns the socio-economic dimensions almost always present outside France [17, 36]. In our study, it had a very little influence on HCV screening, in spite of 17.7% of unemployed, a high rate of respondents outside of the labor force (54.7%), or without a stable housing (45.2%). These differences may be related to the French welfare model, with a universal and free access to OST, and to the rich and diversified supply of free harm reduction programs and healthcare services in Strasbourg.
2. With an overall reported screening rate of 82% and 46% of those found PCR-positive treated, one could consider these results as optimal, much higher than usually reported [7, 13, 37]. However, once analyzed following subpopulations of DUs, it did not appear so positive. First, if 92.6% of DUs attending SDUs had been screened, this rate fell to 75.6% for those who had dropped out and 58.8% for those who had never attended a SDU. Moreover, 7.9% of the screening had been performed more than 1 year before our study. Overall, an optimal screening would have missed 33.9% (95% CI 28.8–39.4) of the recruited DUs. If hepatitis C had been a major concern of the DUs, the screenings would have been the result of active and rational approaches, correlated with past risk-taking, sources of concern about possible contaminations, and a search for information about the effectiveness of treatments. Our data, at odds with this presumption, favor the hypothesis of a moderate interest of the UDs for the hepatitis C inducing little active screening.
  - First, 85.2% of the DUs who had not been screened explained this absence by their lack of concern about HCV hepatitis. Then, we did not find any correlation between the presence of appropriate screening and previous risk-taking. A belief in the efficacy of HCV treatments was present in only 30.1% of those DUs who had not been screened positive, and 42.8% of those who had been screened DUs had no opinion concerning the efficacy of HCV treatments. This limited knowledge is consistent with the literature [17]. At last, the main plans for the future had a concrete and immediate dimension such as work, teeth, and housing. Hepatitis ranked almost last when DUs did not know they were infected. Our study provides elements to understand this lack of correlation:
    We have found a positive impact of a police crackdown on injections on HCV screening, already known to increase contaminations [38, 39]. When one believes, rightly or wrongly, that his usual drug use does not present a specific risk, police interventions, modifying these routines, reveal a risk that has not been internalized and lead to an active hitherto nonexistent decision of screening, even in the absence of a current contact with healthcare providers. As in any educational approach, one is faced with the difficulty of trivializing (internalization of imposed norms) a non-trivial (human) subject [40]. “Being not concerned,” given as the main reason for not having performed HCV tests, may be considered as reflecting a denial, allowing the acceptance of what has become an unconscious contradiction and repressing any conscious thought of an inevitable death [41]. This blindness is favored by the absence of clear messages hierarchizing the risks [16, 17]; if NSS and sharing of works are placed at the same level as sexual intercourse without a condom, sharing sniffing or smoking paraphernalia, toothbrushes, and razors as well as tattoos, is it possible to avoid contamination?
    Fear of contamination that affected almost half of the respondents was independent of the use of drugs (history of injection or NSS), of sexual practices (use of condoms), or of a previous screening. It had only one “medical” association: the project of a future HCV screening. But this plan did not result in a better acceptance of an immediate screening. If drug use was one of its key determinants (strong relationship between fear of contamination and overdoses), other factors came into play such as the total number of fears. It was reduced by a very good social integration and increased by illegal activities. This can be seen as inexplicable with respect to the actual risks of contamination, but from DUs’ point of view, it can be considered as the result of individual “ecological” imaginary constructs reflecting the diversity of their priorities among which the understanding of the message associated with the risks of contamination is part of a global representation of a dangerous lifestyle.
  - If hepatitis B and C screenings, like hepatitis B vaccinations, were not associated with any risk previously taken, they were correlated with attendance to SDUs and, in particular, with the relationship (sources of information, prescription) of DUs with GPs. This finding is in accordance with a passively accepted screening already observed by Jordan et al. [19]. The significant role of the screening proposed systematically at the entry into prison as well as the acceptance of an immediate test by all seronegative DUs whose tests were too old illustrates this passive position and the need to remind professionals of the value of repeating tests when risk-taking persists even if DUs do not require it. In our experience, this passive acceptance, like that of liver biopsies or hepatitis treatments, works often as a reciprocal tradeoff-like exchange where the test (biopsy, treatment) operates, besides and in addition to trust, as a symbolic payment (counter-gift) for the initial gift of the care, provided that its payment is not considered to have been paid in full [42]. Acceptance of screening was favored by the capacity of the professional to provide accurate information on hepatitis B and C and on their treatments as well as on vaccinations as underlined by the centrality of GPs among all health care providers. Observation is already made by Walley and al. [21]. This capacity to explain is mandatory to make the existence of a life-threatening disease credible whose only symptom is most often a non-specific fatigue and of its possible cure [43, 44]. It is the reason for the success of integrated care interventions [45–47] practiced in Strasbourg with an overall rate of treatment higher than usually reported [48, 49].

Ten years later, our assumptions are in line with Treloar and Rhodes’s and expand their conclusion [16]. Justifying our inclusion of non-injectors, we show, for the first time, that there is no difference in this life experience between injectors and non-injectors, confirming the difficulty encountered in understanding and integrating prevention messages which hinder the access to screening and treatment [18].

The low prevalence of HCV and HIV infections observed confirms the trend toward a decline of both incidence already observed in France [32–34, 50] and in other countries [51]. Since we have shown, after others, that risk-taking is not associated with a clear understanding of viral contaminations, this trend cannot be attributed to changes in behavior following a rational risk analysis. It is more likely that the lasting impact induced by the AIDS epidemic on behaviors followed a group mimicry which can be likened to a heuristic strategy [52] known to spread among social networks like epidemics [53–56]. Its impact was favored by access to syringes (in France as early as 1987), OST (in France universal and free access in 1996), and other harm reduction programs where DUs meet care providers. In spite of these positive findings, we can conclude that the control of the HCV epidemic will not come by improving the delivery of targeted prevention message. They will never have the efficacy of the early AIDS messages that had all the characteristics required of a message to perform [57]; it was simple (HIV infection led to death), unexpected (people paid attention), concrete (it was understood and remembered), credible (people agreed and believed), and emotional (people feared) and led people to act (a credible story was told with a simple solution: condoms and sterile works). In addition, access to sterile works was considered satisfying by more than four fifth of injectors and will be difficult to improve significantly. Last, limited access to DAA to the most advanced liver diseases, besides its inefficiency to control the epidemic, maintains the confusion of the messages. Treatment of what is presented as a life-threatening disease would be eligible to only one third of infected UDs if their contamination is old enough to result in a severe liver disease, and, even in that case, the continued use of drugs or alcohol could be sufficient to deny it [13, 58, 59].

## Conclusion

As was proposed for HIV and HCV [60, 61], a universal access to DAA is the best approach to impact significantly the course of the epidemic, provided that most infected DUs are screened and treated for a period short enough to prevent reinfection. We have demonstrated that in a large French city where services are exceptionally developed and free, this objective may not be achieved due to a too small active investment of the DUs. We have to move from a predominant selective screening of DUs attending SDUs to a screening of its entire population. Considering the results of our RDS investigation which is a kind of conditional cash transfer, we believe that this approach could be a solution. With resources much lower than those needed to fund outreach teams, it would be able to recruit UDs who are not interested in SDUs’ offers. The nudge [62, 63] would be the association, that has been effective, of a cash incentive, participation in public health action, and the opportunity to tell one’s story. Providing respondents with an HCV education opportunity, that was not supplied by our study [21], should, with the possible access to an effective treatment through a shared decision [64], improve the rate of acceptance of HCV screenings and treatments. This access, possible in France, removes any concern about the ethical nature of this incentive. This approach would have the additional benefit of improving SDUs through a better knowledge of drug use and DUs’ expectations in the targeted area, and the induced behavioral changes could spread among the DU community through peers’ example [53–56, 65]. Conditional cash transfer has already been successfully used for diverse health programs [66–69]. This hypothesis will need an actual study to be verified.

## Abbreviations

DAA: Direct-acting antiviral
DU: Drug user
NSS: Needle and syringe exchange
OST: Opioid substitution treatment
RDS: Respondent-driven sampling
SDU: Service for DUs
Source: Source of health information
